# A Comparative Study of Integrated Crop Management System vs. Conventional Crop Management System for Cotton Yield and Fiber Quality With Respect to Fruiting Position Under Different Soil Fertility Levels

**DOI:** 10.3389/fpls.2018.00958

**Published:** 2018-08-03

**Authors:** Xinyue Zhang, Hongkun Yang, John L. Snider, Rizwan Zahoor, Babar Iqbal, Binglin Chen, Yali Meng, Zhiguo Zhou

**Affiliations:** ^1^College of Agriculture, Nanjing Agricultural University, Nanjing, China; ^2^Department of Crop and Soil Sciences, University of Georgia, Tifton, GA, United States

**Keywords:** cotton (*Gossypium hirsutum* L.), management system, soil fertility, seedcotton yield, fiber quality, fruiting position

## Abstract

In order to increase cotton productivity and optimize fiber quality on limited arable land, an integrated crop management system (ICMS), which combined with some optimal management practices, is projected to replace the conventional crop management system (CCMS) for cotton production in the Yangtze River valley. The seedcotton yield and fiber quality with respect to fruiting position under ICMS and CCMS were investigated in 2012 and 2013 in two fields differing in soil fertility. Reduced bolls on fruiting branches 1–10 (FB_1−10_) and at fruiting position 1–2 (FP_1−2_) on FB_11−15_ could not be fully compensated by increased bolls on FB_16+_ under CCMS, resulting in more seedcotton yield under ICMS relative to that under CCMS. Fiber at majority fruiting positions under CCMS were longer and stronger than those under ICMS, but CCMS increased the contribution of bolls on FB_11+_ to the cotton yield, which overall resulted in no significant change in fiber length and strength by management system at field level. The number of bolls at FP_1−2_ on FB_1−5_ under CCMS while the number of bolls on FB_1−5_ and at FP_1−2_ on FB_1−5_ were not significantly changed by soil fertility, resulting in diminished yield difference in soil fertility among ICMS relative to that of CCMS. The high soil fertility significantly increased seedcotton yield relative to low soil fertility, which was attributed to more number of bolls on FB_11+_ and higher seedcotton weight per boll at all fruiting positions. High soil fertility field not only recorded superior fiber quality on FB_11+_, but also increased the contribution of these bolls to the cotton yield relative to those in the low soil fertility field, resulting in no significant change in overall fiber quality among soil fertility. These findings demonstrate that by combining optimal management practices on infertile soils ICMS could minimize the yield differences due to soil fertility without sacrificing fiber quality.

## Introduction

Cotton (*Gossypium hirsutum* L.) is an important cash crop and provides raw material for producing textiles. However, with increasing population and greater demand for food in China, a large area of fertile land previously cultivated to cotton has been occupied by food crops (Zhao and Tisdell, 2009). Thus, cotton production has moved to low soil fertility fields (Dong et al., 2012). Low soil fertility has a negative impact on cotton production (Das et al., 2006; Singh and Ahlawat, 2014; Kintché et al., 2015), and this coupled with poor field management have imposed substantial challenges to cotton production (Dong et al., 2009). Therefore, it is essential to optimize management practices to improve seedcotton yield and fiber quality in the low soil fertility fields.

Numerous studies showed that agronomic management practices such as plant density (Bednarz et al., 2000; Ghader et al., 2012; Feng et al., 2014) and nitrogen management (Reddy et al., 2004; Saleem et al., 2010; Yang et al., 2011) have a profound effect on seedcotton yield and fiber quality. Moreover, high crop yields and quality are difficult to achieve by altering single management practices in isolation (Robertson et al., 2000; Ladha et al., 2003), and interactions exist among agronomic practices since yield is determined by the most limiting factor (Dai et al., 2017). An integrated approach that optimizes nutrients and other agronomic management practices would allow the maximization of crop yield (Wang et al., 2017). In order to increase yield, some progressive farmers generally integrate optimized management practices into their conventional crop management system (CCMS); this has been referred to as an integrated crop management system (ICMS) or “best management” system. Several recent reports have indicated that the productivity of rice (Balasubramanian et al., 2005; Cao and Yin, 2015), wheat (Gupta and Seth, 2007) and maize (Jin et al., 2012) are effectively enhanced by adopting ICMS. Whether ICMS can also improve cotton yield and fiber quality requires further study.

Soil fertility is an important factor governing agricultural practices (Sawan et al., 2006). Soil fertility includes levels of organic matter, total nitrogen, available nitrogen, phosphorus, and potassium (Xiong et al., 2003; Zingore et al., 2007). The soil fertility level of a given field can greatly influence crop response to different management practices (CRI, 2013). Dong et al. (2010) found that increased plant density and/or nitrogen rate significantly increased cotton yield in low soil fertility situations but not under high soil fertility. Moreover, soil fertility has different effects on the productivity of cropping systems. Improved soil fertility could increase the yield of long-season cotton or cotton in a double-cropping system, but the yield of short-season cotton or cotton grown in monoculture may not be affected by soil fertility (Feng et al., 2017; Lu et al., 2017). While a number of studies have addressed the impact of management systems or individual management practices on cotton productivity, studies evaluating integrated management practices under contrasting soil fertility levels are limited. This information will be extremely important for producers attempting to make management decisions specifically tailored to their production scenarios.

Cotton produces bolls that vary in size and fruiting position depending on soil quality and other environmental conditions, resulting in different boll distribution patterns and within-canopy fiber quality (Mauney, 1986). Boll distribution patterns and within-canopy fiber quality can be used to explain differences in productivity and to evaluate the effects of crop management (Kerby and Buxton, 1981). Therefore, the objectives of this study were to determine (i) the differences in seedcotton yield and fiber quality with respect to fruiting position under CCMS and ICMS; and (ii) the differential yield responses of CCMS and ICMS to soil fertility; and (iii) how soil fertility influences the seedcotton yield and fiber quality with respect to fruiting position.

## Materials and methods

### Experimental site and plant material

A 2-year field study was conducted during 2012 and 2013 under two different soil fertility levels (low and high soil fertility fields, 200 m apart) at Dafeng, Jiangsu, China (33°19′N, 120°45′E). The soil at the experimental site was typical sandy loam, and two soil fertility levels were identified based on the soil nutrient status (Table 1; Yang et al., 2016). Siza 3, a widely planted cotton cultivar in the Yangtze River Valley was used as plant material. Weather data, i.e. mean daily temperature, mean daily solar radiation and total rainfall during boll development with respect to fruiting position were obtained from a weather station (Campbell AG800, Genetics, USA) located near the experimental site.

**Table 1:** Soil fertility status of the two experimental fields studied in 2012 (*n* = 5).

**Soil fertility**	**Organic matter (g kg^−1^)**	**Total N (g kg^−1^)**	**Available N (mg kg^−1^)**	**Available P (mg kg^−1^)**	**Available K (mg kg^−1^)**
LF	14.33 ± 0.62	0.79 ± 0.04	23.95 ± 1.57	19.04 ± 1.22	364.54 ± 19.35
HF	17.76 ± 0.69	0.86 ± 0.03	28.63 ± 2.02	23.56 ± 0.96	384.06 ± 16.86

*LF, low soil fertility field; HF, high soil fertility field. Data were collected from 0 to 20 cm soil samples before the start of the experiments in 2012*.

### Experimental design

A CCMS and an ICMS was applied to two fields with different soil fertility levels in a 2 × 2 factorial design, with three replicates of the four treatments. CCMS followed a longstanding management practice which is utilized by the majority of farmers in the Yangtze River valley (Yang et al., 2017). The ICMS is based on CCMS but incorporates practices aimed at improving productivity, including optimal plant density, growth-driven fertilizer schedule, simplified seedling rising technology. Notably, the integration of the economic N application rate, number of N splits, and optimum plant density were based on our previous single-factor experiments (Chen et al., 2015, 2016; Meng et al., 2016), and the simplified seedling rising technology for ICMS was aimed to reduce labor cost using mechanized transplantation.

A detailed explanation of each system was provided in Table 2. Nitrogen of CCMS was applied at a rate of 300 kg ha^−1^, with 40% as basal dose and the remaining 60% was applied at the initial flowering stage, whereas nitrogen of ICMS was applied at an economic rate of 375 kg ha^−1^ (calculated according to Baker et al., 2004) in 4 splits, 20% as basal, 25% at the initial flowering stage, 40% at full-bloom stage and the remaining 15% at the end of the flowering stage, which was timed to coincide with growth-driven nitrogen demand of cotton plants. CCMS adopted a labor-intensive traditional seedling rising method (Dong et al., 2007) and seedlings were transplanted manually at a lower density of 18,000 plant ha^−1^, whereas ICMS adopted a simplified seedling rising technology (Dai et al., 2017) and seedlings were transplanted mechanically at an optimal density of 30,000 plants ha^−1^. The cottonseed used in the nursery was treated with fungicides: 8% tolclofos-methyl, 12% thiram, and 6% carbendazim. Seedlings were transplanted in the fields on May 15 when they had 3–4 true leaves. Each plot size was 220 m^2^ with 20 rows, having 1.1 m distance between rows and 0.5 m between plants in the row for CCMS and 0.3 m between plants in the row for ICMS.

**Table 2:** Nutrients management, plant density, and seedling rising method of CCMS and ICMS in 2012 and 2013.

**Management system**	**Fertilizer (kg ha^−1^) (N-P_2_O_5_^†^-K_2_O^†^)**	**N splits (kg N ha^−1^) (A-B-C-D)**	**Management**		
			**Plant density (plant ha^−1^)**	**Plant spacing (m)**	**Seedling rising method**
CCMS	300-300-300	120-180-0-0	18,000	0.5	ST
ICMS	375-225-412	75-94-150-56	30,000	0.3	SR

A, basal fertilizer; B, initial flowering stage; C, full-bloom stage; D, the end of the flowering stage; ST, conventional seedling nursery method. In this method, seeds planted in a nursery bed and then manually transplanted into the field on May 15. SR, Simplified seedling nursery technology. In this method, seeds were planted with bio-organic fertilizer, and mechanically transplanted in to field on May 15.

† *50% of the fertilizer was applied as basal fertilizer and 50% was applied at the initial flowering stage*.

### Sampling and processing

#### Agronomic traits and boll distribution

Vertically, plants were divided into four primary zones, i.e., FB_1−5_ (fruiting branches 1 through 5), FB_6−10_ (fruiting branches 6 through 10), FB_11−15_ (fruiting branches 11 through 15) and FB_16+_ (the 16th and upper fruiting branches). Similarly, plants were divided horizontally into two primary zones, the first and second fruiting positions closest to the main stem (FP_1−2_) and the third and greater fruiting positions (FP_3+_). Fifteen successive plants from three central rows in each replicate plot of two management systems in the low and high soil fertility fields were randomly tagged at maturity to determine the number of fruiting branches and positions, and the number of mature bolls (>2 cm in diameter) on the eight primary zones per unit area.

#### Fiber quality

Bolls from FB_3_, FB_8_, FB_13_ were chosen as representative samples for zones FB_1−5_, FB_6−10_, FB_11−15_, respectively. Bolls from FB_18_ and FB_16_ were chosen to represent FB_16+_ of CCMS and ICMS, respectively. White blooms in each position were tagged with a jeweler tag, labeling the flowering and boll opening date, which encompasses the entire boll development period. All tagged, open bolls from each primary zone were harvested by hand. The seedcotton was ginned, and fiber was sent to the Cotton Quality Supervision, Inspection, and Testing Center of the China Ministry of Agriculture for quality analysis. Fiber qualities including fiber length, strength, and micronaire of each lint sample were measured with the Uster HVI MF100 cotton fiber quality analyzer (USTER®, Uster, Zurich, Switzerland).

### Net photosynthetic rate of the main stem leaves on different fruiting branches

Net photosynthetic rate was measured on main stem leaves of representative fruiting branches on July 15, August 15 and September 15 in 2012 and 2013 in each field. Measurements were made using a Li-6400XT portable photosynthetic system (Li-COR, Lincoln, NE, USA) under 1,500 μmol m^−2^ s^−1^ light intensity, 65 ± 5% relative humidity, 32 ± 2°C leaf temperature and 380 ± 5 ppm CO_2_ between 9:30 and 11:00 a.m. on cloudless days.

### Statistical analyses

The effects of year (Y), soil fertility (S), management system (M) and their interactions on environmental conditions, morphological indices and net photosynthetic rates were analyzed using a full factorial three-way ANOVA. While, The effects of year (Y), soil fertility (S), management system (M), fruiting positions (FP) and their interactions on seedcotton yield, yield components, and fiber quality with respect to fruiting positions were analyzed using a full factorial four-way ANOVA. All statistical analyses in our study were performed using IBM SPSS Statistics 20.0 (SPSS Inc. Chicago, Illinois, USA).

## Results

### Environmental conditions for flowering and boll maturation

The flowering date, time required for boll maturation and the mean daily temperature of bolls with respect to fruiting position varied significantly across years, but they were not affected by the interactions between year and any of other factors (Table 5). The flowering date, time required for boll maturation, and environmental conditions during flowering and boll maturation within a given soil fertility and management system were consistent across years (Table 5).

The flowering date, duration of boll development, and environmental conditions experienced by bolls at different fruiting positions were significantly affected by year and management system, but only the flowering date and time required for boll maturation were significantly affected by soil fertility (Table 5). Among management systems, the average flowering date of bolls with respect to fruiting position under ICMS was delayed 6d and the average time for boll maturation with respect to fruiting position under ICMS was 6d longer relative to bolls under CCMS (Table 3), which resulted in different climate conditions experienced during boll growth and development despite the fact that the bolls were at the same fruiting position for CCMS and ICMS (Table 4). Compared with ICMS, the mean daily temperature during growth and development of bolls under CCMS was elevated by 1.6°C (Table 4A). Among soil fertility levels, low soil fertility delayed the flowering date 4d but shortened the time required for boll maturation by 3d compared with the high soil fertility level. Therefore, there was no significant difference in the mean daily temperature during growth and development of bolls at the same fruiting position between the low and high soil fertility fields (Table 4A).

**Table 3:** Flowering date and time for boll maturation of bolls with respect to fruiting position (FP) under CCMS and ICMS in the low and high soil fertility fields in 2012 and 2013 (*n* = 45).

**Year**	**Fruiting branches**	**Management system**	**FP**_**1−2**_				**FP**_**3+**_			
			**Flowering date (DPS)^†^**		**Time for boll maturation (d)**		**Flowering date (DPS)**		**Time for boll maturation (d)**	
			**LF**	**HF**	**LF**	**HF**	**LF**	**HF**	**LF**	**HF**
2012	FB‡_16+_	CCMS	127.3 ± 1.20	123.4 ± 0.88	54.2 ± 1.20	60.1 ± 1.53	132.4 ± 1.45	128.4 ± 0.88	63.7 ± 1.88	67.0 ± 1.00
		ICMS	133.0 ± 1.53	130.7 ± 1.45	63.6 ± 0.88	68.3 ± 1.20	136.1 ± 1.15	134.1 ± 1.00	68.8 ± 1.88	72.3 ± 0.88
	FB_11−15_	CCMS	119.3 ± 1.67	115.0 ± 1.53	55.0 ± 1.15	58.4 ± 0.67	128.3 ± 1.20	123.0 ± 1.53	59.3 ± 1.20	64.4 ± 1.20
		ICMS	124.6 ± 1.45	122.2 ± 1.20	61.3 ± 1.86	64.0 ± 0.58	132.2 ± 1.20	128.6 ± 0.88	66.1 ± 1.15	70.0 ± 1.15
	FB_6−10_	CCMS	109.7 ± 0.88	106.1 ± 0.88	46.3 ± 0.88	50.1 ± 1.15	124.0 ± 1.15	118.0 ± 1.53	56.0 ± 0.58	59.3 ± 1.76
		ICMS	116.0 ± 1.53	113.8 ± 1.67	53.4 ± 0.88	55.8 ± 1.20	127.2 ± 1.20	122.3 ± 1.20	61.4 ± 1.15	64.0 ± 1.15
	FB_1−5_	CCMS	99.2 ± 0.88	95.0 ± 1.15	40.0 ± 1.53	42.7 ± 0.67	119.3 ± 1.76	113.6 ± 1.20	47.0 ± 1.15	51.2 ± 1.45
		ICMS	103.8 ± 0.88	99.2 ± 1.53	44.5 ± 1.20	47.9 ± 0.88	122.0 ± 1.53	115.7 ± 1.67	53.3 ± 1.20	56.3 ± 1.45
2013	FB_16+_	CCMS	118.1 ± 0.58	115.0 ± 1.15	53.0 ± 0.58	55.3 ± 1.20	122.1 ± 1.00	118.9 ± 1.67	57.4 ± 0.88	63.6 ± 0.88
		ICMS	125.7 ± 1.45	123.3 ± 1.53	59.1 ± 1.00	60.6 ± 1.45	129.0 ± 1.53	126.0 ± 1.53	63.0 ± 1.73	68.8 ± 1.73
	FB_11−15_	CCMS	114.4 ± 1.20	111.1 ± 1.15	48.3 ± 1.20	50.6 ± 0.58	119.6 ± 1.67	117.0 ± 1.15	53.7 ± 2.03	59.3 ± 2.03
		ICMS	123.3 ± 1.20	118.7 ± 1.20	55.2 ± 1.53	56.0 ± 1.45	127.5 ± 0.88	125.3 ± 0.88	59.6 ± 1.76	66.1 ± 1.76
	FB_6−10_	CCMS	104.2 ± 1.15	101.2 ± 1.53	41.4 ± 1.45	43.0 ± 0.88	115.0 ± 1.15	115.3 ± 1.73	51.0 ± 1.53	56.4 ± 1.53
		ICMS	113.0 ± 1.53	109.4 ± 1.76	47.0 ± 0.58	48.2 ± 1.15	123.3 ± 1.76	119.7 ± 1.86	56.3 ± 1.20	61.0 ± 1.20
	FB_1−5_	CCMS	95.3 ± 0.88	91.9 ± 1.67	38.4 ± 1.20	39.3 ± 0.88	111.4 ± 1.00	108.0 ± 1.15	45.5 ± 0.88	47.1 ± 0.88
		ICMS	103.6 ± 1.20	100.0 ± 1.53	42.3 ± 0.88	43.8 ± 1.20	116.0 ± 1.53	113.0 ± 1.53	51.0 ± 1.15	53.3 ± 1.15

*CCMS, conventional crop management system; ICMS, integrated crop management system; LF, low soil fertility field; HF, high soil fertility field*.

† *DPS, days post-sowing*.

^‡^*FB, fruiting branch*.

**Table 4:** Mean daily temperature **(A)**, mean daily solar radiation **(B)**, and total rainfall **(C)** during the period between anthesis and boll open of bolls with respect to fruiting position (FP) under CCMS and ICMS in the low and high soil fertility fields in 2012 and 2013 (*n* = 45).

**Year**	**Fruiting branches**	**Management system**	**FP**_**1−2**_		**FP**_**3+**_	
			**LF**	**HF**	**LF**	**HF**
**(A) MEAN DAILY TEMPERATURE (**°**C)**						
2012	FB^†^_16+_	CCMS	21.5 ± 0.10	21.9 ± 0.05	20.2 ± 0.03	20.5 ± 0.06
		ICMS	20.1 ± 0.03	20.1 ± 0.11	18.9 ± 0.10	19.0 ± 0.13
	FB_11−15_	CCMS	23.2 ± 0.17	23.5 ± 0.07	21.2 ± 0.12	21.7 ± 0.12
		ICMS	21.6 ± 0.15	21.9 ± 0.04	20.1 ± 0.08	20.2 ± 0.11
	FB_6−10_	CCMS	25.4 ± 0.10	25.8 ± 0.13	22.1 ± 0.06	22.8 ± 0.16
		ICMS	23.9 ± 0.12	24.0 ± 0.15	21.1 ± 0.12	22.0 ± 0.10
	FB_1−5_	CCMS	28.1 ± 0.07	28.1 ± 0.03	24.0 ± 0.08	24.4 ± 0.10
		ICMS	26.8 ± 0.14	27.3 ± 0.11	22.7 ± 0.11	23.6 ± 0.16
2013	FB_16+_	CCMS	24.9 ± 0.07	25.5 ± 0.13	24.0 ± 0.06	24.4 ± 0.04
		ICMS	22.4 ± 0.14	23.3 ± 0.12	22.0 ± 0.16	22.5 ± 0.20
	FB_11−15_	CCMS	26.4 ± 0.04	26.7 ± 0.02	24.7 ± 0.08	25.2 ± 0.25
		ICMS	23.7 ± 0.08	24.5 ± 0.08	22.6 ± 0.18	23.1 ± 0.19
	FB_6−10_	CCMS	28.2 ± 0.25	28.8 ± 0.09	25.2 ± 0.11	25.9 ± 0.21
		ICMS	26.6 ± 0.02	27.1 ± 0.11	23.6 ± 0.06	24.3 ± 0.06
	FB_1−5_	CCMS	30.1 ± 0.09	30.5 ± 0.02	27.0 ± 0.05	27.3 ± 0.05
		ICMS	28.1 ± 0.13	28.8 ± 0.29	25.7 ± 0.18	26.5 ± 0.12
**(B) MEAN DAILY SOLAR RADIATION (MJ m**^−2^**)**						
2012	FB^†^_16+_	CCMS	15.3 ± 0.03	14.9 ± 0.10	14.3 ± 0.12	14.5 ± 0.14
		ICMS	14.3 ± 0.09	14.2 ± 0.10	13.7 ± 0.03	13.6 ± 0.02
	FB_11−15_	CCMS	14.9 ± 0.01	14.5 ± 0.00	15.0 ± 0.05	14.8 ± 0.04
		ICMS	15.0 ± 0.05	14.7 ± 0.01	14.2 ± 0.11	14.1 ± 0.09
	FB_6−10_	CCMS	14.4 ± 0.07	15.2 ± 0.10	15.1 ± 0.03	14.7 ± 0.03
		ICMS	14.2 ± 0.07	14.6 ± 0.08	15.0 ± 0.02	14.9 ± 0.05
	FB_1−5_	CCMS	17.2 ± 0.03	17.3 ± 0.03	14.5 ± 0.07	14.3 ± 0.07
		ICMS	15.7 ± 0.11	17.0 ± 0.09	14.9 ± 0.02	14.4 ± 0.04
2013	FB_16+_	CCMS	15.5 ± 0.08	15.9 ± 0.02	14.9 ± 0.18	15.4 ± 0.16
		ICMS	13.9 ± 0.08	14.6 ± 0.06	13.8 ± 0.05	13.9 ± 0.09
	FB_11−15_	CCMS	16.2 ± 0.11	16.4 ± 0.03	15.4 ± 0.04	15.6 ± 0.06
		ICMS	14.6 ± 0.17	15.2 ± 0.02	14.0 ± 0.12	14.4 ± 0.09
	FB_6−10_	CCMS	18.0 ± 0.22	18.3 ± 0.18	15.3 ± 0.09	16.1 ± 0.19
		ICMS	16.7 ± 0.02	16.8 ± 0.07	14.5 ± 0.13	15.2 ± 0.16
	FB_1−5_	CCMS	18.2 ± 0.22	18.6 ± 0.13	16.6 ± 0.07	16.6 ± 0.05
		ICMS	18.0 ± 0.11	18.5 ± 0.19	15.8 ± 0.11	16.3 ± 0.11
**(C) TOTAL RAINFALL (mm)**						
2012	FB^†^_16+_	CCMS	149.6 ± 0.00	152.1 ± 0.87	121.9 ± 5.32	157.2 ± 3.87
		ICMS	128.5 ± 6.08	128.8 ± 0.20	148.7 ± 6.67	149.1 ± 0.00
	FB_11−15_	CCMS	155.1 ± 0.00	158.7 ± 0.00	150.0 ± 0.93	154.9 ± 0.93
		ICMS	152.2 ± 0.93	156.9 ± 0.77	128.6 ± 2.32	175.7 ± 0.17
	FB_6−10_	CCMS	163.5 ± 0.00	163.5 ± 0.00	149.9 ± 0.00	155.5 ± 0.00
		ICMS	158.6 ± 0.00	158.7 ± 0.00	152.4 ± 0.07	154.3 ± 0.90
	FB_1−5_	CCMS	68.8 ± 1.73	68.8 ± 0.00	155.1 ± 0.00	158.7 ± 0.00
		ICMS	141.5 ± 4.87	125.9 ± 18.18	154.3 ± 0.00	158.6 ± 0.00
2013	FB_16+_	CCMS	89.2 ± 0.00	89.2 ± 0.00	111.6 ± 14.90	98.0 ± 5.67
		ICMS	129.4 ± 0.17	134.4 ± 0.17	128.5 ± 0.00	134.4 ± 0.00
	FB_11−15_	CCMS	81.6 ± 5.81	81.6 ± 0.47	89.2 ± 0.00	89.2 ± 2.53
		ICMS	133.9 ± 14.90	111.6 ± 2.93	129.4 ± 0.17	134.4 ± 0.17
	FB_6−10_	CCMS	104.6 ± 2.50	103.0 ± 2.50	89.2 ± 2.53	81.6 ± 7.18
		ICMS	63.5 ± 0.00	64.2 ± 0.00	133.9 ± 11.97	111.6 ± 12.90
	FB_1−5_	CCMS	111.7 ± 8.50	97.2 ± 4.33	63.5 ± 0.00	100.7 ± 0.00
		ICMS	104.6 ± 0.53	103.0 ± 2.50	81.6 ± 2.53	81.6 ± 6.03

*CCMS, conventional crop management system; ICMS, integrated crop management system; LF, low soil fertility field; HF, high soil fertility field*.

† *FB, fruiting branch*.

**Table 5:** Results of ANOVA on the effects of year (Y), soil fertility (S), management system (M), and their interactions on time for boll maturation, mean daily temperature, mean daily solar radiation, and total rainfall during the period between anthesis and boll open of bolls with respect to fruiting position.

**Effect**	***df***	**Flowering date**	**Time for boll maturation**	**Mean daily temperature**	**Mean daily solar radiation**	**Total rainfall**
Year	1	13.875^**^	36.655^**^	68.191^**^	47.321^**^	172.883^**^
Soil fertility	1	6.239^*^	6.103^*^	2.698	0.867	0.699
Management system	1	19.076^**^	30.557^*^	17.710^**^	19.113^**^	9.400^**^
Y × S	1	0.144	1.109	0.330	1.489	1.987
Y × M	1	0.820	0.069	0.086	3.707	4.345^*^
S × M	1	0.008	0.041	0.167	0.051	0.224
Y × S × M	1	0.077	0.015	0.077	0.000	0.068

F-values and significance levels (^^**^^P < 0.01, ^^*^^P < 0.05). df, degree of freedom.

### Morphological indices of cotton

All the morphological indices of cotton measured in the present study varied significantly across years, but there was no interaction between year and any other effects (Table 6). The variations in morphological indices with soil fertility and management system were consistent across years (Table 6).

**Table 6:** Effects of management system on morphological indices in the low and high soil fertility fields in 2012 and 2013 (*n* = 45).

**Soil fertility**	**Year**	**Management system**	**Fruiting branches number (no. plant^−1^)**	**Fruiting positions number (no. plant^−1^)**	**Ratio of fruiting positions to fruiting branches^†^**	**Fruiting branches number (10^4^ no. ha^−1^)**	**Fruiting positions number (10^4^ no. ha^−1^)**	**Boll retention (%)**
LF	2012	CCMS	19.67 ± 0.17	101.29 ± 1.54	5.15 ± 0.06	35.40 ± 0.30	182.32 ± 2.78	38.10 ± 0.72
		ICMS	15.80 ± 0.21	71.09 ± 1.21	4.50 ± 0.06	47.40 ± 0.64	213.27 ± 3.63	36.51 ± 0.71
	2013	CCMS	20.60 ± 0.27	115.33 ± 2.51	5.59 ± 0.08	37.08 ± 0.48	207.60 ± 4.52	37.89 ± 0.46
		ICMS	17.18 ± 0.19	84.91 ± 1.68	4.94 ± 0.08	51.53 ± 0.57	254.73 ± 5.05	35.61 ± 0.44
HF	2012	CCMS	20.33 ± 0.20	119.82 ± 2.21	5.89 ± 0.09	36.60 ± 0.36	215.44 ± 3.98	37.76 ± 0.46
		ICMS	16.11 ± 0.24	82.11 ± 1.23	5.11 ± 0.05	48.33 ± 0.71	246.33 ± 3.70	32.46 ± 0.58
	2013	CCMS	21.56 ± 0.25	131.80 ± 1.52	6.12 ± 0.05	38.80 ± 0.45	237.24 ± 2.73	37.60 ± 0.43
		ICMS	17.40 ± 0.18	92.67 ± 1.52	5.33 ± 0.08	52.20 ± 0.53	278.00 ± 4.57	34.39 ± 0.52
**SIGNIFICANCE**								
Year (Y)			62.649^**^	33.845^**^	46.532^**^	64.997^**^	115.434^**^	0.058
Soil fertility (S)			12.518^**^	38.576^**^	134.628^**^	9.409^**^	114.206^**^	4.789^*^
Management system (M)			661.257^**^	254.622^**^	215.822^**^	1225.556^**^	179.230^**^	21.097^**^
Y × S			0.108	0.326	3.601	0.030	1.466	1.138
Y × M			0.704	0.044	0.008	3.817	2.544	0.266
S × M			3.194	3.768	2.107	0.802	0.356	2.984
Y × S × M			0.384	0.009	0.000	0.285	0.296	1.059

*CCMS, conventional crop management system; ICMS, integrated crop management system; LF, low soil fertility field; HF, high soil fertility field*.

† *Ratio of fruiting positions to fruiting branches, fruiting positions/fruiting branches*.

F-values and significance levels (^**^P < 0.01, ^*^P < 0.05).

The morphological indices of cotton were significantly affected by soil fertility and management system but no S × M interaction was observed (Table 6). Among management systems, the number of fruiting branches, and fruiting positions per plant, the ratio of fruiting positions to fruiting branches, and boll retention under ICMS were significantly lower than those under CCMS. Conversely, the number of fruiting branches and fruiting positions per hectare under ICMS were higher than those of CCMS due to different plant densities in the contrasting management systems. Whether assessed on a per plant or per hectare basis, among soil fertility levels, the number of fruiting branches, fruiting positions, and the ratio of fruiting positions to fruiting branches under the two management systems in the low soil fertility field were lower than those in the high soil fertility field; however, boll retention exhibited an inverse trend.

### Seedcotton yield and yield component distribution within the canopy

Seedcotton yield, boll number and seedcotton weight per boll with respect to fruiting position varied significantly across years, but no interactions between year and any other effect were observed (Table 8). The variations in seedcotton yield and yield components with respect to fruiting position, soil fertility, and management system were consistent across years (Table 8).

The seedcotton yield, boll number and seedcotton weight per boll at a given fruiting position were significantly affected by soil fertility, management system (except seedcotton weight per boll), and M × FP interaction, but not by S × M and S × FP interactions (Table 8). Among management systems, ICMS produced more bolls on FB_1−10_ and at FP_1−2_ on FB_11−15_ than CCMS. In contrast, the number of bolls on FB_16+_ under ICMS was significantly lower than for the CCMS treatment (Table 7A). Interestingly, a differential response of boll distribution to fertility levels was observed under management systems. Compared with the management systems in the high soil fertility field, the number of bolls at FP_1−2_ on FB_1−5_ under CCMS was not significantly changed by decreased soil fertility, while the number of bolls on FB_1−5_ and at FP_1−2_ on FB_6−10_ under ICMS were not significantly changed by decreased soil fertility. With respect to soil fertility, the number of bolls on FB_11+_ in the low soil fertility field was significantly reduced relative to that in the high soil fertility field. Similar results were found in seedcotton yield with respect to fruiting position (Table 7C), and seedcotton weight per boll at all fruiting positions was decreased in the low soil fertility field relative to that in the high soil fertility field (Table 7B).

**Table 7:** Effects of management system on boll number **(A)**, seedcotton weight per boll **(B)**, seedcotton yield **(C)**, and contribution rate to yield **(D)** of boll with respect to fruiting position (FP) in the low and high soil fertility fields in 2012 and 2013 (*n* = 45).

**Year**	**Fruiting branches**	**Management system**	**FP**_**1−2**_		**FP**_**3+**_	
			**LF**	**HF**	**LF**	**HF**
**(A) BOLL NUMBER (10**^4^ **no. ha**^−1^**)**						
2012	FB^†^_16+_	CCMS	6.11 ± 0.78	7.32 ± 0.92	4.51 ± 0.61	6.23 ± 0.61
		ICMS	2.68 ± 0.87	4.12 ± 1.00	1.01 ± 0.13	2.49 ± 0.58
	FB_11−15_	CCMS	7.31 ± 0.95	8.61 ± 0.90	8.12 ± 0.58	9.81 ± 0.58
		ICMS	10.00 ± 0.60	11.21 ± 0.64	6.93 ± 0.53	8.89 ± 0.52
	FB_6−10_	CCMS	10.10 ± 0.46	10.78 ± 0.50	12.43 ± 0.51	13.91 ± 0.51
		ICMS	15.59 ± 0.58	16.01 ± 0.55	14.90 ± 0.55	16.20 ± 0.53
	FB_1−5_	CCMS	10.10 ± 0.53	10.31 ± 0.52	10.81 ± 0.57	11.83 ± 0.58
		ICMS	14.90 ± 0.53	15.00 ± 0.53	12.66 ± 0.57	12.90 ± 0.51
2013	FB_16+_	CCMS	7.31 ± 0.60	8.57 ± 0.55	7.90 ± 0.50	10.21 ± 0.50
		ICMS	4.32 ± 1.00	5.62 ± 0.95	3.21 ± 0.67	4.72 ± 0.68
	FB_11−15_	CCMS	10.02 ± 0.55	10.90 ± 0.53	10.80 ± 0.61	12.68 ± 0.68
		ICMS	12.53 ± 0.75	13.10 ± 0.85	10.21 ± 1.03	12.42 ± 1.03
	FB_6−10_	CCMS	10.81 ± 0.58	11.72 ± 0.53	13.02 ± 0.58	14.58 ± 0.58
		ICMS	16.70 ± 0.59	16.51 ± 0.60	16.10 ± 0.60	17.30 ± 0.60
	FB_1−5_	CCMS	10.62 ± 0.61	10.90 ± 0.53	8.40 ± 0.60	9.51 ± 0.59
		ICMS	15.50 ± 0.76	15.61 ± 0.85	11.30 ± 0.53	11.10 ± 0.55
**(B) SEEDCOTTON WEIGHT PER BOLL (g)**						
2012	FB^†^_16+_	CCMS	5.14 ± 0.04	5.24 ± 0.03	4.77 ± 0.04	5.08 ± 0.04
		ICMS	4.95 ± 0.02	4.97 ± 0.09	4.84 ± 0.10	4.90 ± 0.11
	FB_11−15_	CCMS	5.51 ± 0.02	5.72 ± 0.06	5.37 ± 0.06	5.47 ± 0.05
		ICMS	5.44 ± 0.07	5.49 ± 0.02	5.36 ± 0.05	5.37 ± 0.06
	FB_6−10_	CCMS	6.14 ± 0.06	6.42 ± 0.01	5.97 ± 0.01	6.24 ± 0.04
		ICMS	6.30 ± 0.02	6.48 ± 0.01	6.10 ± 0.06	6.30 ± 0.06
	FB_1−5_	CCMS	5.36 ± 0.05	5.58 ± 0.01	5.20 ± 0.02	5.40 ± 0.01
		ICMS	5.58 ± 0.05	5.68 ± 0.03	5.33 ± 0.02	5.46 ± 0.02
2013	FB_16+_	CCMS	4.79 ± 0.08	5.10 ± 0.03	4.62 ± 0.02	4.90 ± 0.02
		ICMS	4.65 ± 0.04	4.94 ± 0.08	4.45 ± 0.05	4.81 ± 0.05
	FB_11−15_	CCMS	5.18 ± 0.05	5.50 ± 0.07	4.93 ± 0.01	5.30 ± 0.03
		ICMS	4.96 ± 0.02	5.40 ± 0.05	4.78 ± 0.02	5.20 ± 0.01
	FB_6−10_	CCMS	5.54 ± 0.05	5.70 ± 0.02	5.37 ± 0.04	5.50 ± 0.03
		ICMS	5.59 ± 0.06	5.80 ± 0.01	5.44 ± 0.02	5.70 ± 0.01
	FB_1−5_	CCMS	5.10 ± 0.07	5.25 ± 0.05	4.89 ± 0.04	5.11 ± 0.05
		ICMS	5.16 ± 0.02	5.30 ± 0.03	4.96 ± 0.09	5.20 ± 0.02
**(C) SEEDCOTTON YIELD (kg ha**^−1^**)**						
2012	FB^†^_16+_	CCMS	314.40 ± 40.93	376.49 ± 49.11	214.81 ± 30.14	315.24 ± 32.15
		ICMS	134.11 ± 43.59	203.99 ± 50.08	49.20 ± 6.35	121.19 ± 30.44
	FB_11−15_	CCMS	402.23 ± 51.51	484.42 ± 52.52	435.00 ± 32.15	536.15 ± 32.15
		ICMS	544.22 ± 30.55	615.40 ± 37.86	370.42 ± 29.69	478.01 ± 30.55
	FB_6−10_	CCMS	610.13 ± 30.00	693.22 ± 32.79	741.31 ± 32.15	867.10 ± 31.79
		ICMS	983.45 ± 34.64	1037.30 ± 36.17	909.34 ± 33.29	1021.02 ± 32.15
	FB_1−5_	CCMS	541.00 ± 28.87	575.31 ± 29.87	562.33 ± 30.44	637.32 ± 31.22
		ICMS	831.89 ± 30.14	852.02 ± 30.55	676.52 ± 30.55	704.25 ± 29.67
2013	FB_16+_	CCMS	350.29 ± 30.00	439.34 ± 30.00	364.64 ± 22.55	500.00 ± 25.03
		ICMS	200.06 ± 47.01	276.78 ± 48.54	141.75 ± 28.31	226.02 ± 32.15
	FB_11−15_	CCMS	518.00 ± 30.00	600.27 ± 30.55	533.11 ± 30.55	672.64 ± 33.29
		ICMS	620.40 ± 40.00	707.19 ± 47.26	488.06 ± 49.24	645.19 ± 54.08
	FB_6−10_	CCMS	598.28 ± 32.15	667.23 ± 31.15	697.99 ± 32.15	803.20 ± 32.15
		ICMS	934.17 ± 32.15	957.10 ± 33.05	867.18 ± 35.12	986.32 ± 35.12
	FB_1−5_	CCMS	541.21 ± 30.55	573.44 ± 30.35	411.07 ± 30.14	484.98 ± 30.00
		ICMS	800.09 ± 40.34	827.21 ± 45.09	557.28 ± 30.00	577.23 ± 30.11
**(D) CONTRIBUTION RATE TO YIELD (%)**						
2012	FB^†^_16+_	CCMS	8.16 ± 0.55	8.43 ± 0.62	5.58 ± 0.37	6.99 ± 0.30
		ICMS	2.91 ± 0.82	4.08 ± 0.78	1.08 ± 0.09	2.36 ± 0.48
	FB_11−15_	CCMS	10.45 ± 0.60	10.74 ± 0.50	11.38 ± 0.20	11.96 ± 0.15
		ICMS	12.09 ± 0.07	12.22 ± 0.31	8.20 ± 0.24	9.49 ± 0.16
	FB_6−10_	CCMS	16.02 ± 0.36	15.49 ± 0.24	19.47 ± 0.54	19.30 ± 0.51
		ICMS	21.89 ± 0.47	20.65 ± 0.61	20.24 ± 0.37	20.33 ± 0.49
	FB_1−5_	CCMS	14.20 ± 0.39	12.84 ± 0.29	14.74 ± 0.26	14.23 ± 0.20
		ICMS	18.52 ± 0.32	16.96 ± 0.29	15.06 ± 0.08	14.01 ± 0.26
2013	FB_16+_	CCMS	8.69 ± 0.25	9.05 ± 0.17	9.09 ± 0.05	10.55 ± 0.01
		ICMS	4.25 ± 0.70	5.56 ± 0.58	3.04 ± 0.44	4.31 ± 0.38
	FB_11−15_	CCMS	12.90 ± 0.10	12.66 ± 0.18	13.28 ± 0.26	14.08 ± 0.22
		ICMS	13.45 ± 0.06	13.59 ± 0.26	10.55 ± 0.48	12.47 ± 0.33
	FB_6−10_	CCMS	14.92 ± 0.46	13.89 ± 0.21	17.41 ± 0.28	17.09 ± 0.28
		ICMS	20.35 ± 0.63	18.45 ± 0.47	18.87 ± 0.42	18.81 ± 0.48
	FB_1−5_	CCMS	13.48 ± 0.05	12.45 ± 0.09	10.22 ± 0.22	10.42 ± 0.12
		ICMS	17.39 ± 0.23	15.91 ± 0.15	12.10 ± 0.13	11.10 ± 0.10

*CCMS, conventional crop management system; ICMS, integrated crop management system; LF, low soil fertility field; HF, high soil fertility field*.

† *FB, fruiting branch*.

The contributions of bolls at different fruiting positions to yield was significantly affected by S × FP and M × FP interactions (Table 8). Bolls on upper fruiting branches (FB_11+_) were more important contributors to overall yield production under CCMS and/or in the high soil fertility field than those of ICMS and/or in the low soil fertility field. The average contribution of the bolls on FB_11+_ to the total yield under CCMS was 11.13% higher than that under ICMS and the average contribution of the bolls on FB_11+_ to the total yield in the high soil fertility field was 3.48% higher than that in the low soil fertility field (Table 7D).

**Table 8:** Results of ANOVA on the effects of year (Y), soil fertility (S), management system (M), fruiting position (FP) and their interactions on boll number, seedcotton weight per boll, seedcotton yield, and contribution rate to yield of boll with respect to fruiting position.

**Effect**	***df***	**Boll number**	**Seedcotton weight per boll**	**Seedcotton yield**	**Contribution rate to yield**
Year	1	59.731^**^	163.244^**^	6.746^*^	0.000
Soil fertility	1	42.805^**^	118.448^**^	79.981^**^	0.000
Management system	1	46.381^**^	0.884	65.801^**^	0.000
Fruiting position	7	231.050^**^	402.988^**^	321.564^**^	1326.471^**^
Y × S	1	0.013	0.652	0.191	0.000
Y × M	1	0.023	3.242	0.369	0.006
Y × FP	7	12.531^**^	7.062^**^	15.468^**^	73.184^**^
S × M	1	0.915	0.741	0.860	0.001
S × FP	7	1.931	0.364	1.826	14.765^**^
M × FP	7	58.700^**^	3.849^**^	63.447^**^	204.886^**^
Y × S × M	1	0.247	2.278	0.000	0.000
Y × S × FP	7	0.073	0.384	0.170	0.299
Y × M × FP	7	0.600	1.315	0.558	3.460^**^
S × M × FP	7	0.220	0.569	0.213	1.348
Y × S × M × FP	7	0.022	2.081	0.036	0.142

F-values and significance levels (^**^P < 0.01, ^*^P < 0.05). df, degree of freedom.

### Fiber quality distribution within the canopy

The fiber quality parameters length, strength and micronaire were significantly affected by year, soil fertility, management system, and fruiting position (Table 10).

Fiber length and strength were also significantly affected by Y × M, M × FP, and Y × M × FP interactions (Table 10). The effects of management system on fiber length and strength differed in different years and fruiting branches (Table 10). In 2012, the fiber of bolls at FP_1−2_ on FB_1−5_ under ICMS was longer and stronger than that under CCMS, and an inverse trend was detected at all other fruiting positions in either field. While, in 2013, the fiber of bolls at FP_1−2_ on FB_1−5_ under ICMS was longer and stronger than fiber produced under CCMS; however, an inverse trend was only detected at FP_3+_ on FB_6−10_ and on FB_11+_ in either field (Tables 9A,B). By comparison, the micronaire was not significantly affected by Y × M, M × FP, and Y × M × FP interactions (Table 10). Fiber micronaire at all fruiting positions under ICMS was consistently lower than under CCMS in 2012 and 2013 in either field (Table 9C).

**Table 9:** Effects of management system on fiber length **(A)**, strength **(B)**, and micronaire **(C)** of boll with respect to fruiting position in the low and high soil fertility fields in 2012 and 2013 (*n* = 18).

**Year**	**Fruiting branches**	**Management system**	**FB**_**1−2**_		**FB**_**3+**_	
			**LF**	**HF**	**LF**	**HF**
**(A) FIBER LENGTH (mm)**						
2012	FB^†^_16+_	CCMS	27.89 ± 0.18	28.68 ± 0.15	26.83 ± 0.16	27.79 ± 0.26
		ICMS	26.81 ± 0.23	27.82 ± 0.15	26.03 ± 0.10	26.82 ± 0.26
	FB_11−15_	CCMS	29.52 ± 0.20	30.68 ± 0.17	28.62 ± 0.15	29.54 ± 0.21
		ICMS	28.74 ± 0.23	29.59 ± 0.10	27.48 ± 0.13	28.48 ± 0.14
	FB_6−10_	CCMS	32.38 ± 0.21	32.54 ± 0.12	30.72 ± 0.16	31.59 ± 0.12
		ICMS	31.48 ± 0.20	31.67 ± 0.16	29.71 ± 0.10	30.20 ± 0.24
	FB_1−5_	CCMS	28.18 ± 0.15	27.81 ± 0.20	29.90 ± 0.18	30.24 ± 0.36
		ICMS	29.98 ± 0.28	30.30 ± 0.17	29.70 ± 0.17	29.17 ± 0.20
2013	FB_16+_	CCMS	27.50 ± 0.10	28.29 ± 0.17	26.49 ± 0.14	27.42 ± 0.36
		ICMS	26.21 ± 0.15	27.52 ± 0.10	25.39 ± 0.20	26.42 ± 0.18
	FB_11−15_	CCMS	29.99 ± 0.17	30.88 ± 0.15	28.11 ± 0.21	28.91 ± 0.14
		ICMS	29.11 ± 0.12	29.88 ± 0.16	27.27 ± 0.12	28.20 ± 0.18
	FB_6−10_	CCMS	31.63 ± 0.19	31.83 ± 0.15	30.49 ± 0.21	31.34 ± 0.22
		ICMS	31.11 ± 0.22	31.23 ± 0.20	29.62 ± 0.20	30.09 ± 0.16
	FB_1−5_	CCMS	28.24 ± 0.15	27.90 ± 0.13	28.97 ± 0.31	29.21 ± 0.18
		ICMS	29.83 ± 0.11	30.17 ± 0.19	29.32 ± 0.10	29.41 ± 0.24
**B FIBER STRENGTH (g tex**^−1^**)**						
2012	FB^†^_16+_	CCMS	28.07 ± 0.25	28.98 ± 0.13	26.96 ± 0.09	27.75 ± 0.22
		ICMS	27.14 ± 0.28	27.96 ± 0.14	25.71 ± 0.17	26.92 ± 0.11
	FB_11−15_	CCMS	30.58 ± 0.16	31.62 ± 0.19	29.48 ± 0.16	30.83 ± 0.19
		ICMS	29.59 ± 0.17	30.32 ± 0.10	28.28 ± 0.18	29.68 ± 0.30
	FB_6−10_	CCMS	33.04 ± 0.21	33.57 ± 0.14	32.03 ± 0.12	32.78 ± 0.17
		ICMS	31.85 ± 0.18	32.25 ± 0.13	30.92 ± 0.11	31.30 ± 0.18
	FB_1−5_	CCMS	29.97 ± 0.16	29.77 ± 0.30	31.26 ± 0.12	31.63 ± 0.12
		ICMS	31.53 ± 0.13	31.81 ± 0.20	30.19 ± 0.19	30.59 ± 0.20
2013	FB_16+_	CCMS	29.47 ± 0.15	30.24 ± 0.11	28.44 ± 0.16	29.27 ± 0.32
		ICMS	28.56 ± 0.23	29.18 ± 0.19	27.15 ± 0.08	28.17 ± 0.14
	FB_11−15_	CCMS	32.13 ± 0.22	33.06 ± 0.14	31.02 ± 0.19	31.94 ± 0.11
		ICMS	31.56 ± 0.33	32.18 ± 0.10	30.09 ± 0.27	31.02 ± 0.09
	FB_6−10_	CCMS	33.88 ± 0.29	34.21 ± 0.35	32.63 ± 0.31	33.48 ± 0.20
		ICMS	34.18 ± 0.27	34.20 ± 0.24	31.52 ± 0.17	32.06 ± 0.17
	FB_1−5_	CCMS	30.52 ± 0.22	30.28 ± 0.43	31.73 ± 0.08	32.05 ± 0.26
		ICMS	32.63 ± 0.09	32.97 ± 0.20	31.93 ± 0.03	31.73 ± 0.14
**(C) MICRONAIRE (units)**						
2012	FB^†^_16+_	CCMS	4.31 ± 0.08	4.60 ± 0.04	4.11 ± 0.11	4.31 ± 0.09
		ICMS	3.91 ± 0.07	4.20 ± 0.07	3.52 ± 0.09	3.99 ± 0.14
	FB_11−15_	CCMS	4.90 ± 0.04	5.01 ± 0.04	4.62 ± 0.09	4.61 ± 0.14
		ICMS	4.49 ± 0.06	4.70 ± 0.05	4.20 ± 0.03	4.31 ± 0.16
	FB_6−10_	CCMS	5.19 ± 0.03	5.30 ± 0.09	4.79 ± 0.04	4.91 ± 0.05
		ICMS	4.99 ± 0.12	5.01 ± 0.02	4.56 ± 0.09	4.60 ± 0.05
	FB_1−5_	CCMS	5.19 ± 0.04	5.39 ± 0.09	4.92 ± 0.11	4.90 ± 0.06
		ICMS	4.91 ± 0.12	5.10 ± 0.04	4.60 ± 0.05	4.69 ± 0.07
2013	FB_16+_	CCMS	4.61 ± 0.05	4.89 ± 0.11	4.40 ± 0.10	4.62 ± 0.10
		ICMS	4.30 ± 0.06	4.52 ± 0.04	3.99 ± 0.09	4.21 ± 0.08
	FB_11−15_	CCMS	5.09 ± 0.10	5.21 ± 0.05	4.82 ± 0.13	5.01 ± 0.08
		ICMS	4.80 ± 0.06	5.01 ± 0.02	4.40 ± 0.07	4.69 ± 0.14
	FB_6−10_	CCMS	5.41 ± 0.10	5.39 ± 0.12	5.21 ± 0.08	5.20 ± 0.08
		ICMS	5.00 ± 0.10	5.00 ± 0.04	4.80 ± 0.09	4.90 ± 0.10
	FB_1−5_	CCMS	5.50 ± 0.09	5.39 ± 0.12	5.20 ± 0.10	5.22 ± 0.08
		ICMS	5.20 ± 0.11	5.30 ± 0.07	4.91 ± 0.10	4.91 ± 0.10

*CCMS, conventional crop management system; ICMS, integrated crop management system; LF, low soil fertility field; HF, high soil fertility field*.

† *FB, fruiting branch*.

**Table 10:** Results of ANOVA on the effects of year (Y), soil fertility (S), management system (M), fruiting position (FP) and their interactions on fiber length, strength, and micronaire of boll with respect to fruiting position.

**Effect**	***df***	**Fiber length**	**Fiber strength**	**Micronaire**
Year	1	31.984^**^	602.530^**^	142.383^**^
Soil fertility	1	137.442^**^	153.813^**^	37.888^**^
Management system	1	95.614^**^	119.797^**^	230.580^**^
Fruiting position	7	544.645^**^	703.391^**^	147.327^**^
Y × S	1	0.099	2.561	0.819
Y × M	1	4.503^*^	19.636^**^	0.020
Y × FP	7	4.872^**^	6.658^**^	1.978
S × M	1	0.002	0.216	1.602
S × FP	7	9.907^**^	7.488^**^	2.542^*^
M × FP	7	58.207^**^	60.753^**^	0.946
Y × S × M	1	0.827	0.309	0.007
Y × S × FP	7	0.241	0.454	0.822
Y × M × FP	7	2.148^*^	3.649^**^	0.528
S × M × FP	7	1.969	1.235	0.263
Y × S × M × FP	7	0.224	0.131	0.422

F-values and significance levels (^**^P < 0.01, ^*^P < 0.05). df, degree of freedom.

Fiber length, strength, and micronaire were also significantly affected by S × FP interaction (Table 10). Among soil fertility, there was no significant difference in fiber quality on FB_1−10_ between the low and high soil fertility fields, but fiber length, strength, and micronaire on FB_11+_ in the low soil fertility field were lower than those in the high soil fertility field (Table 9).

### Net photosynthetic rate of the main stem leaves on different fruiting branches

On August 15, the net photosynthetic rate of leaves on FB_1−5_, FB_6−10_, and FB_11−15_ was significantly affected by year, soil fertility, and management system, but only the net photosynthetic rate of leaves on FB_1−5_ was significantly affected by S × M interaction (Table 11).

**Table 11:** Effects of management system on net photosynthetic rate of the main stem leaves on different fruiting branches (FB) in the low and high soil fertility fields in 2012 and 2013 (*n* = 27).

**Year**	**Soil fertility**	**Management system**	**Net photosynthetic rate (**μ **mol m**^**−2**^ **s**^**−1**^**)**							
			**15-Jul**	**15-Aug**			**15-Sep**			
			**FB^†^_1−5_**	**FB_1−5_**	**FB_6−10_**	**FB_11−15_**	**FB_1−5_**	**FB_6−10_**	**FB_11−15_**	**FB_16+_**
2012	LF	CCMS	28.39 ± 0.39	14.31 ± 0.38	19.29 ± 0.32	20.90 ± 0.24	4.99 ± 0.11	6.71 ± 0.23	8.69 ± 0.21	10.19 ± 0.30
		ICMS	28.12 ± 0.47	15.99 ± 0.32	21.48 ± 0.27	23.31 ± 0.21	5.60 ± 0.22	9.01 ± 0.21	11.99 ± 0.18	13.91 ± 0.22
	HF	CCMS	29.45 ± 0.39	16.10 ± 0.20	21.01 ± 0.21	24.29 ± 0.19	4.90 ± 0.36	7.90 ± 0.25	10.41 ± 0.27	11.91 ± 0.22
		ICMS	29.09 ± 0.39	16.51 ± 0.31	23.02 ± 0.19	25.40 ± 0.25	3.90 ± 0.23	8.61 ± 0.36	13.01 ± 0.20	15.51 ± 0.27
2013	LF	CCMS	29.59 ± 0.29	14.50 ± 0.21	19.39 ± 0.26	22.40 ± 0.35	4.73 ± 0.19	6.90 ± 0.16	9.00 ± 0.18	10.41 ± 0.26
		ICMS	29.50 ± 0.29	16.30 ± 0.13	22.00 ± 0.19	23.79 ± 0.18	5.19 ± 0.18	9.11 ± 0.21	12.39 ± 0.37	14.41 ± 0.26
	HF	CCMS	30.70 ± 0.28	15.94 ± 0.25	21.20 ± 0.25	24.70 ± 0.40	4.90 ± 0.21	8.01 ± 0.09	10.81 ± 0.20	12.50 ± 0.29
		ICMS	30.49 ± 0.33	16.63 ± 0.46	23.41 ± 0.29	26.03 ± 0.32	3.72 ± 0.21	8.50 ± 0.13	13.61 ± 0.19	15.70 ± 0.21
**SIGNIFICANCE**										
Year (Y)			25.883^**^	0.226	9.308^**^	14.721^**^	2.141	0.333	6.770^*^	6.800^*^
Soil fertility (S)			16.952^**^	22.690^**^	125.440^**^	160.806^**^	33.666^**^	6.277^*^	76.089^**^	133.047^**^
Management system (M)			0.929	29.747^**^	257.295^**^	62.242^**^	4.726^*^	120.694^**^	335.164^**^	628.272^**^
Y × S			0.001	0.520	0.652	1.415	0.669	0.292	0.193	0.013
Y × M			0.135	0.273	0.654	1.012	0.529	0.334	0.193	0.045
S × M			0.062	7.885^*^	0.390	3.030	34.542^**^	40.842^**^	3.801	2.485
Y × S × M			0.000	0.045	1.468	2.422	2.141	0.057	0.021	1.364

*CCMS, conventional crop management system; ICMS, integrated crop management system; LF, low soil fertility field; HF, high soil fertility field*.

† *FB, fruiting branch*.

F-values and significance levels (^**^P < 0.01, ^*^P < 0.05).

The net photosynthetic rate of leaves on FB_6−15_ under the two management systems in the low soil fertility field was lower than that in the high soil fertility field. But there was no significant difference under ICMS between the low and high soil fertility fields, while the net photosynthetic rate of leaves on FB_1−5_ under CCMS in the low soil fertility field was lower than in the high soil fertility field (Table 11).

On September 15, the net photosynthetic rate of leaves on FB_1−5_, FB_6−10_, FB_11−15_, and FB_16+_ was significantly affected by year (except FB_1−5_ and FB_6−10_), soil fertility, and management system, but only the net photosynthetic rate of leaves on FB_1−5_ and FB_6−10_ was significantly affected by S × M interaction (Table 11).

The net photosynthetic rate of leaves on FB_11+_ under the two management systems in the low soil fertility field was lower than that in the high soil fertility field. However, there was no significant difference in the net photosynthetic rate of leaves on FB_6−10_ under ICMS between the low and high soil fertility fields, and the net photosynthetic rate of leaves on FB_6−10_ under CCMS in the low soil fertility field was lower than that in the high soil fertility field. Moreover, the net photosynthetic rate of leaves on FB_1−5_ under ICMS in the low soil fertility field was higher than that in the high soil fertility field and there was no significant difference in the net photosynthetic rate of leaves on FB_1−5_ under CCMS between the low and high soil fertility fields (Table 11).

## Discussion

### Seedcotton yield and fiber quality of boll with respect to fruiting position under CCMS and ICMS

The fruiting habit of the cotton plant is altered by nitrogen rate and plant density, where plants produced more bolls on upper branches and produce more horizontal fruiting sites along longer fruiting branches as plant density is decreased (Bednarz et al., 2000) and nitrogen rate is increased (Boquet et al., 1994). In our study, despite having lower nitrogen rates and split applications, CCMS allowed the plants to set more apical fruiting branches and distal fruiting positions than did the plants under ICMS, which had a higher plant density (Table 6). Reduced plant density in cotton allowed for yield compensation by increasing boll production on upper branches (Bednarz et al., 2000). In our study, the seedcotton yield of bolls on FB_16+_ played a compensating role in yield production under CCMS. Boll reduction on FB_1−10_ and at FP_1−2_ on FB_11−15_ under CCMS was not compensated for by bolls formed at FP_3+_ on FB_11−15_. Moreover, this loss could not be fully compensated for by increased bolls on FB_16+_ and led to more total bolls produced under ICMS relative to CCMS in either field (Table 7A). This resulted in yield differences between CCMS and ICMS that were mainly associated with plant density differences between the two management systems.

Fiber length, strength and micronaire were significantly affected by management system. Fiber micronaire at all fruiting positions under ICMS were consistently higher than those under CCMS, which was in agreement with the previous research that micronaire was increased with increasing levels of nitrogen application under high temperature and reduced by increased nitrogen application under low temperature (Boman et al., 1997). Fiber length and strength were significantly affected by management system × fruiting position interaction (Table 10). The fiber at FP_1−2_ on FB_1−5_ under ICMS was longer and stronger than that of CCMS in either field due to the fact that more nitrogen fertilizer was applied at the initial flowering stage under CCMS (Tables 9A,B). This result is in agreement with (Zhao et al., 2012), where high temperature during the boll development period along with a high nitrogen rate was not conducive to fiber quality formation. 23~25°C was the optimal temperature for fiber quality formation (Reddy et al., 1999) and supplemented nitrogen fertilizer could mitigate the negative influence of low temperature on fiber quality in the later flowering season (Reddy et al., 2004; Zhao et al., 2012). However, in our study, although the ICMS applied nitrogen at the end of the flowering stage, the fiber on upper fruiting branches under ICMS was shorter and weaker than those of CCMS. Lower temperature decreased sucrose export in cotton leaves (Liu et al., 2013) and less photosynthate was transferred to fiber, which resulted in inferior fiber quality (Liu et al., 2015a,b). This may be due to the temperature during boll growth under ICMS, which was 1~2°C lower than for CCMS (Table 4A), and the positive effect of nitrogen application was likely masked by the temperature difference. These results indicated that fiber length, strength and micronaire, was not just a function of management but also depended on the climatic conditions prevailing during boll development. Interestingly, there was no significant difference in fiber length and strength between CCMS and ICMS at the field level, regardless of year and soil fertility (data not shown). Though fiber length and strength at many fruiting positions under CCMS were superior to ICMS, the seedcotton yield of bolls on upper fruiting branches supported a higher percentage of the total seedcotton yield under CCMS. In comparison, the bolls on upper fruiting branches had inferior fiber quality to those on the lower fruiting branches (Table 9), in agreement with previous studies (Knight et al., 1988; Crawley et al., 1996). Hence, there was no significant difference in fiber length and strength between CCMS and ICMS at the field level in either field. These results indicated that we could achieve higher seedcotton yield without limiting fiber quality by applying integrated crop management practices by enhancing the number of bolls on the fruiting branches and positions where fiber quality is generally superior.

Climatic extremes such as drought and/or elevated temperature become more frequent with climate change (Gilgen and Feller, 2013; Mittal et al., 2014), which potentially negatively affect nutrient availability and plant growth (Aragon and De Datta, 1982; Feller and Vaseva, 2014; He and Dijkstra, 2014). Drought reduced available soil nitrogen and then reduced the number of bolls on upper fruiting branches (Wang et al., 2016). The cotton yield loss of a drought-stressed cotton crop could be compensated for by optimizing nitrogen rate and postponing part of nitrogen fertilization through enhancing water use efficiency (Li et al., 2017; Shi et al., 2018). The ICMS utilized in the current study utilized a growth stage-driven fertilizer schedule which ensured and adequate nitrogen supply for plant growth, which may mitigate the impact of drought on seedcotton yield compared with CCMS. Elevated temperature also adversely affects fiber quality on lower fruiting branches under CCMS but it improves fiber quality on upper fruiting branches under ICMS, which may result in superior fiber quality for ICMS relative to CCMS in extreme high-temperature events. Therefore, maybe ICMS has a greater capacity to tolerate extreme climate events than CCMS.

### The differential yield response of CCMS and ICMS to soil fertility

The boll number per hectare under ICMS and CCMS in the low soil fertility field were 8.0 and 11.7% less than those in the high soil fertility field, respectively (Table 7A), which resulted in reduced seedcotton yield difference in soil fertility among ICMS relative to that of CCMS.

Plant growth was adversely affected by low soil fertility and both CCMS and ICMS exhibited fewer upper fruiting branches and number of nodes per fruiting branch in the low soil fertility field than in the high soil fertility field (Table 6). However, there was a differential response of the boll number and seedcotton yield to decreased soil fertility in response to management system (Tables 7A,C). The plants under CCMS with a low plant density had larger inter-plant space to exploit (Eaton, 1955), and less organic and inorganic nutrients supplied in the low soil fertility field that reduced the net photosynthetic rate of the leaves (Table 11), thus resulting in reduced source strength and capacity to retain bolls at FP_3+_ on FB_1−5_ and on FB_6+_ under CCMS in the low soil fertility field relative to the high soil fertility field. However, the plants under ICMS with a high plant density had less inter-plant space for growth, and the increased leaf area index expected by improving soil fertility would reduce the efficiency of photosynthetic photon flux density interception (Heitholt, 1994) and decrease net photosynthetic rate of leaves on lower fruiting branches (FB_1−10_) (Table 11). Decreased soil fertility reduced the net photosynthetic rate of the leaves on FB_11+_ but increased soil fertility enhanced the net photosynthetic rate of the leaves on FB_1−10_ under ICMS. Therefore, more assimilate production of leaves on lower fruiting branches due to increased net photosynthetic rate of leaves on FB_1−10_ (Buxton et al., 1977) in the low soil fertility field led to more boll retention on lower fruiting branches under ICMS relative to the high soil fertility field. Finally, the number of bolls on FB_1−5_ and at FP_1−2_ on FB_6−10_ under ICMS was not significantly changed by decreased soil fertility. Our results indicate that the yield difference due to soil fertility was lower for ICMS because it combined some optimal management practices relative to those of CCMS; so ICMS could mitigate the impact of cotton production on low fertility soils.

### Seedcotton yield and fiber quality of boll with respect to fruiting position in the low and high soil fertility fields

The nutrient status of the high soil fertility field contributed to a greater number of bolls (Table 7A) and higher seedcotton weight per boll (Table 7B), which was consistent with prior reports (Feng et al., 2017). Furthermore, the results suggested that low soil fertility would decrease the number of bolls on upper fruiting branches and seedcotton weight per boll at all fruiting positions, resulting in lower yield of the low soil fertility field relative to those in the high soil fertility field.

There was no significant difference in fiber length, strength and micronaire between the low and high soil fertility fields at the field level; similar results were observed by Blaise et al. (2005), who reported that fiber length, strength and micronaire were not significantly affected by the application of farmyard manure. However, we found that fiber length, strength, and micronaire of the bolls on the upper fruiting branches in the low soil fertility field were inferior to those in the high soil fertility field. This is because the high soil fertility field could maintain a constant supply of soil nutrients and maintain net photosynthetic rate of leaves on upper fruiting branches, thereby supporting a greater boll load and improving fiber quality (Pettigrew, 1995). In addition, a greater percentage of the total yield was attributed to upper fruiting branches in the high soil fertility field, where fiber quality is generally inferior (Table 9). Thus, the net effect was no measurable gain in overall fiber length, strength and micronaire between the low and high soil fertility fields.

## Conclusions

In our study, we evaluated the responses of seedcotton yield and fiber quality as a function of fruiting positions, management systems, and soil fertility levels. Our results clearly demonstrated that:

- The boll reduction on FB_1−10_ and at FP_1−2_ on FB_11−15_ under CCMS could not be fully compensated for by increased bolls on FB_16+_, resulting in lower overall seedcotton yield under CCMS relative to that under ICMS.
- The number of bolls at FP_1−2_ on FB_1−5_ under CCMS while the number of bolls on FB_1−5_ and at FP_1−2_ on FB_6−10_ under ICMS were not significantly changed by soil fertility, resulting in diminished yield differences in soil fertility among ICMS relative to that of CCMS.
- High soil fertility enhanced seedcotton yield by increasing the number of bolls on upper fruiting branches (FB_11+_) and the seedcotton weight per boll at all fruiting positions.
- Fiber length and strength at the majority of fruiting positions under CCMS was superior to those under ICMS. However, there was no significant difference in fiber length and strength between CCMS and ICMS at the field level, which was attributed to the elevated contribution of bolls on lower fruiting branches (FB_1−10_) to the total yield.
- High soil fertility increased not only fiber length, strength, and micronaire, but also the contribution of bolls on upper fruiting branches (FB_11+_) to the total yield. Thus, fiber length, strength and micronaire were not changed by soil fertility at the field level.
- ICMS maybe have a greater capacity to tolerate extreme climate events than CCMS, but this needs to be further verified in the future.

## Author contributions

ZZ, YM, and BC conceived the idea and led the study design. XZ carried out the experiment, performed analysis and wrote the paper. HY assisted in plant sampling and laboratory analysis. RZ, JS and BI assisted manuscript writing and editing. All authors reviewed the manuscript.

### Conflict of interest statement

The authors declare that the research was conducted in the absence of any commercial or financial relationships that could be construed as a potential conflict of interest.

## References

[B1] Aragon E. De Datta S. (1982). Drought response of rice at different nitrogen levels using line source. Irrigat. Sci. 3, 63–73. 10.1007/BF00264850

[B2] Baker D. A. Young D. L. Huggins D. R. Pan W. L. (2004). Economically optimal nitrogen fertilization for yield and protein in hard red spring wheat. Agron. J. 96, 116–123. 10.2134/agronj2004.0116

[B3] Balasubramanian V. Rajendran R. Ravi V. Chellaiah N. Castro E. Chandrasekaran B. (2005). Integrated crop management for enhancing yield, factor productivity and profitability in Asian rice farms. IRC Newsl. 54, 63–73.

[B4] Bednarz C. W. Bridges D. C. Brown S. M. (2000). Analysis of cotton yield stability across population densities. Agron. J. 92, 128–135. 10.2134/agronj2000.921128x

[B5] Blaise D. Singh J. Bonde A. Tekale K. Mayee C. (2005). Effects of farmyard manure and fertilizers on yield, fibre quality and nutrient balance of rainfed cotton (*Gossypium hirsutum*). Bioresour. Technol. 96, 345–349. 10.1016/j.biortech.2004.03.008 15474936

[B6] Boman R. Raun W. Westerman R. Banks J. (1997). Long-term nitrogen fertilization in short-season cotton: interpretation of agronomic characteristics using stability analysis. J. Produc. Agric. 10, 580–585. 10.2134/jpa1997.0580

[B7] Boquet D. J. Moser E. B. Breitenbeck G. A. (1994). Boll weight and within-plant yield distribution in field-grown cotton given different levels of nitrogen. Agron. J. 86, 20–26. 10.2134/agronj1994.00021962008600010005x

[B8] Buxton D. Briggs R. Patterson L. Watkins S. (1977). Canopy characteristics of narrow-row cotton as influenced by plant density. Agron. J. 69, 929–933. 10.2134/agronj1977.00021962006900060009x

[B9] Cao Y. Yin B. (2015). Effects of integrated high-efficiency practice versus conventional practice on rice yield and N fate. Agric. Ecosyst. Environ. 202, 1–7. 10.1016/j.agee.2015.01.001

[B10] Chen B. Yang H. Song W. Liu C. Xu J. Zhao W. (2016). Effect of N fertilization rate on soil alkali-hydrolyzable N, subtending leaf N concentration, fiber yield, and quality of cotton. Crop J. 4, 323–330. 10.1016/j.cj.2016.03.006

[B11] Chen M. Zhao W. Meng Y. Chen B. Wang Y. Zhou Z. (2015). A model for simulating the cotton (*Gossypium hirsutum* l.) embryo oil and protein accumulation under varying environmental conditions. Field Crops Res. 183, 79–91. 10.1016/j.fcr.2015.07.011

[B12] Crawley S. R. Jenkins J. N. McCarty J. C. (1996). Comparison of fiber properties by fruiting positions of two cultivars, in Proceedings of Beltwide Cotton Production Conference, (Nashville, TN).

[B13] CRI (2013). Cultivation of Cotton in China. Shanghai: Shanghai Science and Technology Press.

[B14] Dai J. Kong X. Zhang D. Li W. Dong H. (2017). Technologies and theoretical basis of light and simplified cotton cultivation in China. Field Crops Res. 214, 142–148. 10.1016/j.fcr.2017.09.005

[B15] Das A. Prasad M. Gautam R. Shivay Y. (2006). Productivity of cotton (*Gossypium hirsutum*) as influenced by organic and inorganic sources of nitrogen. Indian J. Agri. Sci. 76, 354–357.

[B16] Dong H. Kong X. Li W. Tang W. Zhang D. (2010). Effects of plant density and nitrogen and potassium fertilization on cotton yield and uptake of major nutrients in two fields with varying fertility. Field Crops Res. 119, 106–113. 10.1016/j.fcr.2010.06.019

[B17] Dong H. Li W. Eneji A. E. Zhang D. (2012). Nitrogen rate and plant density effects on yield and late-season leaf senescence of cotton raised on a saline field. Field Crops Res. 126, 137–144. 10.1016/j.fcr.2011.10.005

[B18] Dong H. Li W. Tang W. Li Z. Zhang D. (2007). Enhanced plant growth, development and fiber yield of Bt transgenic cotton by an integration of plastic mulching and seedling transplanting. Ind. Crop Prod. 26, 298–306. 10.1016/j.indcrop.2007.03.008

[B19] Dong H. Li W. Tang W. Zhang D. (2009). Early plastic mulching increases stand establishment and lint yield of cotton in saline fields. Field Crops Res. 111, 269–275. 10.1016/j.fcr.2009.01.001

[B20] Eaton F. M. (1955). Physiology of the cotton plant. Ann. Rev. Plant Physiol. 6, 299–328. 10.1146/annurev.pp.06.060155.001503

[B21] Feller U. Vaseva I. I. (2014). Extreme climatic events: impacts of drought and high temperature on physiological processes in agronomically important plants. Front. Environ. Sci. 2:39 10.3389/fenvs.2014.00039

[B22] Feng L. Mathis G. Ritchie G. Han Y. (2014). Optimizing irrigation and plant density for improved cotton yield and fiber quality. Agron. J. 106, 1111–1118. 10.2134/agronj13.0503

[B23] Feng L. Wang G. Han Y. Li Y. Zhu Y. Zhou Z. (2017). Effects of planting pattern on growth and yield and economic benefits of cotton in a wheat-cotton double cropping system versus monoculture cotton. Field Crops Res. 213, 100–108. 10.1016/j.fcr.2017.07.003

[B24] Ghader F. F. Aali M. S. M. Cancholi O. Yousefidaz M. Miri A. A. (2012). Yield and Fiber Quality Comparison of Cotton Planted in Ultra-narrow Row and Conventional Row. 75–91.

[B25] Gilgen A. K. Feller U. (2013). Drought stress alters solute allocation in broadleaf dock (*Rumex obtusifolius*). Weed Sci. 61, 104–108. 10.1614/WS-D-12-00074.1

[B26] Gupta R. Seth A. (2007). A review of resource conserving technologies for sustainable management of the rice–wheat cropping systems of the Indo-Gangetic plains (IGP). Crop Prot. 26, 436–447. 10.1016/j.cropro.2006.04.030

[B27] He M. Dijkstra F. A. (2014). Drought effect on plant nitrogen and phosphorus: a meta-analysis. New Phytologist. 204, 924–931. 10.1111/nph.12952 25130263

[B28] Heitholt J. J. (1994). Canopy characteristics associated with deficient and excessive cotton plant population densities. Crop Sci. 34, 1291–1297. 10.2135/cropsci1994.0011183X003400050028x

[B29] Jin L. Cui H. Li B. Zhang J. Dong S. Liu P. (2012). Effects of integrated agronomic management practices on yield and nitrogen efficiency of summer maize in North China. Field Crops Res. 134, 30–35. 10.1016/j.fcr.2012.04.008

[B30] Kerby T. Buxton D. (1981). Competition between adjacent fruiting forms in cotton. Agron. J. 73, 867–871. 10.2134/agronj1981.00021962007300050028x

[B31] Kintché K. Guibert H. Sogbedji J. Levêque J. Bonfoh B. Tittonell P. (2015). Long-term mineral fertiliser use and maize residue incorporation do not compensate for carbon and nutrient losses from a Ferralsol under continuous maize–cotton cropping. Field Crops Res. 184, 192–200. 10.1016/j.fcr.2015.04.019

[B32] Knight B. Jenkins J. N. McCarty J. C. (1988). Effects of fruiting position on field and fiber quality in commercial cotton, in Proceedings of Beltwide Cotton Production Conference, (New Orleans, LA).

[B33] Ladha J. K. Hill J. E. Duxbury J. M. Gupta R. K. Buresh R. J. (2003). Improving the Productivity and Sustainability of Rice-Wheat Systems: Issues and Impacts. American Society of Agronomy-Crop Science Society of America-Soil Science Society of America, Madison, WI.

[B34] Li W. Li Y. Feng S. (2017). Regulation of root-sourced ABA to growth and water use efficiency of cottonseedling and their response to different nitrogen levels and distribution ratios. Acta Eco. Sin. 37, 6712–6723 (In Chinese, with English abstract).

[B35] Liu J. Ma Y. Lü F. Chen J. Zhou Z. Wang Y. Oosterhuis, D. M. (2013). Changes of sucrose metabolism in leaf subtending to cotton boll under cool temperature due to late planting. Field Crops Res. 144, 200–211. 10.1016/j.fcr.2013.02.003

[B36] Liu J. Meng Y. Chen J. Lü F. Ma Y. Chen B. Oosterhuis D. M. (2015a). Effect of late planting and shading on cotton yield and fiber quality formation. Field Crops Res. 183, 1–13. 10.1016/j.fcr.2015.07.008

[B37] Liu J. Meng Y. Lü F. Chen J. Ma Y. Wang Y.. (2015b). Photosynthetic characteristics of the subtending leaf of cotton boll at different fruiting branch nodes and their relationships with lint yield and fiber quality. Front. Plant Sci. 6:747. 10.3389/fpls.2015.00747 26442060 PMC4584985

[B38] Lu H. Dai J. Li W. Tang W. Zhang D. Eneji A. E. (2017). Yield and economic benefits of late planted short-season cotton versus full-season cotton relayed with garlic. Field Crops Res. 200, 80–87. 10.1016/j.fcr.2016.10.006

[B39] Mauney J. R. (1986). Vegetative growth and development of fruiting sites, in Cotton Physiology, eds Mauney J. R. Stewart J. M. (Memphis, TN: The Cotton Foundation), 11–28.

[B40] Meng Y. Lv F. Zhao W. Chen J. Zhu L. Wang Y. (2016). Plant density influences fiber sucrose metabolism in relation to cotton fiber quality. Acta physiol. Plant. 38:112 10.1007/s11738-016-2129-3

[B41] Mittal N. Mishra A. Singh R. Kumar P. (2014). Assessing future changes in seasonal climatic extremes in the Ganges river basin using an ensemble of regional climate models. Clim. Change 123, 273–286. 10.1007/s10584-014-1056-9

[B42] Pettigrew W. T. (1995). Source-to-sink manipulation effects on cotton fiber quality. Agron. J. 87, 947–952. 10.2134/agronj1995.00021962008700050029x

[B43] Reddy K. R. Davidonis G. H. Johnson A. S. Vinyard B. T. (1999). Temperature regime and carbon dioxide enrichment alter cotton boll development and fiber properties. Agron. J. 91, 851–858. 10.2134/agronj1999.915851x

[B44] Reddy K. R. Koti S. Davidonis G. H. Reddy V. R. (2004). Interactive effects of carbon dioxide and nitrogen nutrition on cotton growth, development, yield, and fiber quality. Agron. J. 96, 1148–1157. 10.2134/agronj2004.1148

[B45] Robertson G. P. Paul E. A. Harwood R. R. (2000). Greenhouse gases in intensive agriculture: contributions of individual gases to the radiative forcing of the atmosphere. Science. 289, 1922–1925. 10.1126/science.289.5486.1922 10988070

[B46] Saleem M. Maqsood M. Javaid A. Hassan M. Khaliq T. (2010). Optimum irrigation and integrated nutrition improves the crop growth and net assimilation rate of cotton (*Gossipium hirsutum* L.). Pak. J. Bot. 42, 3659–3669.

[B47] Sawan Z. Mahmoud M. El-Guibali A. H. (2006). Response of yield, yield components, and fiber properties of Egyptian cotton (*Gossypium barbadense* L.) to nitrogen fertilization and foliar-applied potassium and mepiquat chloride. J. Cotton Sci. 10, 224–234.

[B48] Shi H. Zhang J. Yan Q. Li C. Li J. (2018). Compensation effects of nitrogen fertilizer on yield and quality cotton under insufficient irrigation. J. Plant Nutr. Fertilizers. 24, 134–145 (In Chinese, with English abstract). 10.11674/zwyf.17090

[B49] Singh R. J. Ahlawat I. (2014). Effects of transgenic cotton-based cropping systems and their fertility levels on succeeding wheat crop. Commun. Soil Sci. Plant. 45, 2385–2396. 10.1080/00103624.2014.912291

[B50] Wang D. Huang J. Nie L. Wang F. Ling X. Cui K.. (2017). Integrated crop management practices for maximizing grain yield of double-season rice crop. Sci. Rep. 7:8982. 10.1038/srep38982 28079051 PMC5227689

[B51] Wang R. Ji S. Zhang P. Meng Y. Wang Y. Chen B. (2016). Drought effects on cotton yield and fiber quality on different fruiting branches. Crop Sci. 56, 1265–1276. 10.2135/cropsci2015.08.0477

[B52] Xiong H. Tang Y. Ren D. Li X. Cheng K. Yao W. (2003). Studies on relationships between different soil types and climate condition and grains yield of rice. Southwest China, J. Agric. Sci. 17, 305–309 (In Chinese, with English abstract). 10.3969/j.issn.10014829.2004.04.009

[B53] Yang G. Tang H. Nie Y. Zhang X. (2011). Responses of cotton growth, yield, and biomass to nitrogen split application ratio. Eur. J. Agron. 35, 164–170. 10.1016/j.eja.2011.06.001

[B54] Yang H. Meng Y. Chen B. Zhang X. Wang Y. Zhao W.. (2016). How integrated management strategies promote protein quality of cotton embryos: high levels of soil available N, N assimilation and protein accumulation rate. Front. Plant Sci. 7:1118. 10.3389/fpls.2016.01118 27532007 PMC4969568

[B55] Yang H. Zhang X. Chen B. Meng Y. Wang Y. Zhao W.. (2017). Integrated management strategies increase cottonseed, oil and protein poduction: the key role of carbohydrate metabolism. Front Plant Sci. 8:48. 10.3389/fpls.2017.00048 28194156 PMC5277014

[B56] Zhao W. Wang Y. Zhou Z. Meng Y. Chen B. Oosterhuis D. M. (2012). Effect of nitrogen rates and flowering dates on fiber quality of cotton (*Gossypium hirsutum* L.). Am. J. Exp. Agr. 2:133 10.9734/AJEA/2012/954

[B57] Zhao X. Tisdell C. (2009). The sustainability of cotton production in china and in Australia: comparative economic and environment issues. Clement Allan Tisdell. 19, 265–289.

[B58] Zingore S. Murwira H. K. Delve R. J. Giller K. E. (2007). Influence of nutrient management strategies on variability of soil fertility, crop yields and nutrient balances on smallholder farms in Zimbabwe. Agr. Ecosyst. Environ. 119, 112–126. 10.1016/j.agee.2006.06.019

